# Influence of Elevation Data Resolution on Spatial Prediction of Colluvial Soils in a Luvisol Region

**DOI:** 10.1371/journal.pone.0165699

**Published:** 2016-11-15

**Authors:** Vít Penížek, Tereza Zádorová, Radka Kodešová, Aleš Vaněk

**Affiliations:** Department of Soil Science and Soil Protection, Faculty of Agrobiology, Food and Natural Resources, Czech University of Life Sciences Prague, Prague, Czech Republic; University of Missouri Columbia, UNITED STATES

## Abstract

The development of a soil cover is a dynamic process. Soil cover can be altered within a few decades, which requires updating of the legacy soil maps. Soil erosion is one of the most important processes quickly altering soil cover on agriculture land. Colluvial soils develop in concave parts of the landscape as a consequence of sedimentation of eroded material. Colluvial soils are recognised as important soil units because they are a vast sink of soil organic carbon. Terrain derivatives became an important tool in digital soil mapping and are among the most popular auxiliary data used for quantitative spatial prediction. Prediction success rates are often directly dependent on raster resolution. In our study, we tested how raster resolution (1, 2, 3, 5, 10, 20 and 30 meters) influences spatial prediction of colluvial soils. Terrain derivatives (altitude, slope, plane curvature, topographic position index, LS factor and convergence index) were calculated for the given raster resolutions. Four models were applied (boosted tree, neural network, random forest and Classification/Regression Tree) to spatially predict the soil cover over a 77 ha large study plot. Models training and validation was based on 111 soil profiles surveyed on a regular sampling grid. Moreover, the predicted real extent and shape of the colluvial soil area was examined. In general, no clear trend in the accuracy prediction was found without the given raster resolution range. Higher maximum prediction accuracy for colluvial soil, compared to prediction accuracy of total soil cover of the study plot, can be explained by the choice of terrain derivatives that were best for Colluvial soils differentiation from other soil units. Regarding the character of the predicted Colluvial soils area, maps of 2 to 10 m resolution provided reasonable delineation of the colluvial soil as part of the cover over the study area.

## Introduction

The delineation of soil classes based on digital soil mapping (DSM) using auxiliary data has developed extensively in last two decades. It has been applied at local, regional [1] [2], national [3] [4] [5] or at global scales [6] [7]. Development of these methods is either connected to mapping of new areas [6] or to updating of legacy data such as traditional soil maps [8] [9] [10] [11].

The soils cover evolution is a dynamic process, occurring over a long period. However, the human influence very often leads to an intensification of one or more of the soil forming processes, including soil erosion [12]. Erosion processes change formerly homogenous soil cover into a soil mosaic with high heterogeneity. It is caused by a truncation of soil profiles in sloping and convex parts of the landscape, and an increase of soil thickness due to sedimentation in concave parts of the landscape [13] [14]. Typically, the soil cover of a certain area is stratified into a mosaic of original untouched soil units, degraded soils with truncated profiles (Regosols) and Colluvial soils in slope bases where a large amount of eroded material accumulates, and often fully buries the original soils [12] [15]. Colluvial soils are recognised as an important soil unit because they are a vast sink of soil organic carbon. Colluvial soils very often fill the side valleys as well and form prolongated areas in the soil mosaic [16]. This spatial arrangement of colluvial soil in the landscape resembles the spatial character of Fluvisols or Gleysols in valley bottoms around water courses, characterized by prolongated narrow polygons [17]. Such soil cover change can develop within a few tens of years, which necessitates soil map updating [5] [18] [19].

Soil cover development is traditionally recognized as a result of a set of soil forming factors, defined by Dokuchayev and Jenny [20], and quantitatively redefined by McBratney as the SCORPAN model [21]. The influence of the single soil forming factors varies. At a local scale, the topography is the main soil forming factor that influences the local soil properties and units variability [22]. This is enhanced in areas where erosion processes play an important role. The terrain derivatives, calculated from digital elevation models (DEMs), are then used as the only predictors. Such an approach is used in many studies dealing with soil units spatial delineation [1] [2]. Terrain derivatives quantitatively characterize the shape of the terrain (primary derivatives—e.g. slope gradient, slope aspect, profile and contour curvature) or connected processes (secondary derivatives—e.g. topographic position index or convergence index). The terrain derivatives used as auxiliary data vary within the studies and there is no general model that is applied [22].

In soil terrain modelling, several aspects should be considered: choice of appropriate terrain derivatives, choice of models/methods and spatial scale of the modelling given by raster resolution.

In DSM, the determination of an optimal grid size for prediction of soil properties using environmental factors is still an unsolved issue with only a few empirical guidelines available [23]. The spatial resolution of the DEMs is driven by several factors, such as the resolution of the primary data, computational resources, spatial extent of the mapped areas or demanded resolution of the final map product. The resolution of DEMs varies among studies, from sub-meter resolution to a resolution of hundreds of meters. Many of the regional or global studies use a resolution of 90 meters, which is given by an easily accessible SRTM global DEM product [24]. Local studies use a wide range of DEM sources, such as interpolated contours [25], stereographic photographs or LIDAR data.

The raster resolution directly influences the computation of terrain derivatives and can significantly change the morphological description of the study area [26] [27] [28] [29]. On one hand, the range of many terrain derivatives decreases (slope gradients tend to decrease, ranges in curvatures decrease, flow-path lengths) with increasing pixel size due to DEM flattening [30] [25] [31]. On the other hand, an increase of DEM grid size shifts the topographic index towards higher values due to greater upslope contributing area and smaller slope [32] [33] [34] [35]. The general expectation that DEMs with highest resolution deliver the best results is not always valid [30] [36] [37]. Some studies showed that the prediction accuracy increases with increasing raster resolution at a pixel range of hundreds to tens of meters [38] [39] [40], but this trend is often not significant when the pixel size ranges from a few meters to 1 meter or even sub-meter resolution. This can be explained by the fact that at finer resolutions, terrain attributes hold an excess of details generating too much “noise” that, inevitably invalidate the accuracy of the prediction [23]. In some cases, the optimal resolution is driven by the character (size and shape) of the mapped terrain features [41].

### Background and aim of study

Up-to-date soil maps are important sources of information for agricultural or environmental applications and modelling. The agriculture land of the Czech Republic was mapped at the scale 1:10 000 in 1960s. These maps represent a vast source of spatial information, but during the last 50 years, the soil cover has changed significantly due to intensive soil erosion as a consequence of land consolidation. We believe that our study represents a useful background in the actualization of this map source, namely in regions impacted by soil mass redistribution due to soil erosion.

The purpose of this study is to test the role of spatial scale and its impact on colluvial soil spatial prediction by testing the interaction between pixel resolution (1, 2, 3, 5, 10, 20 and 30m) and prediction accuracy. The specific objective of our study was to examine the influence of pixel resolution on (i) general success rate of applied models, (ii) performance of the models with regard to the spatial extent of predicted colluvial soils and (iii) shape of delineated areas of colluvial soils.

## Material and Methods

### Regional setting

**Fig 1:**
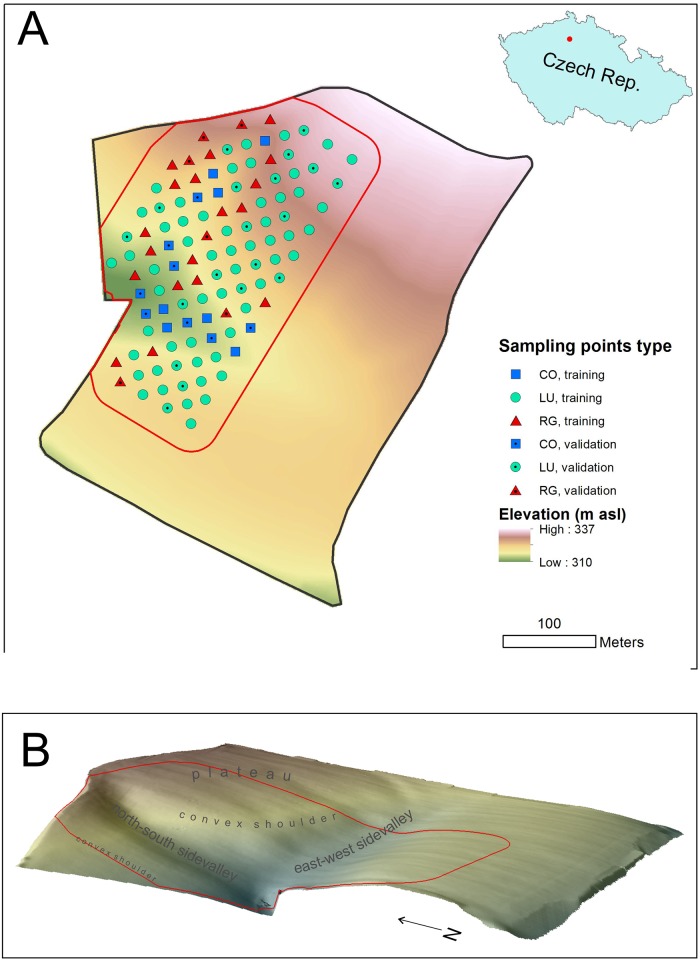
Study plot with sampling scheme. A. The study plot (77 ha) is a part of a larger agricultural plot (176 ha), it is located in the Northern part of the Czech Republic. Soil survey with regular soil sampling scheme was preceded and the sampling points were randomly divided to training and validation subsets. B. Geomorphologic features of the study plot (vertical scale is 2.5x to horizontal scale). The plot is formed by two perpendicular side valleys and a plateau. The transition between these two units is characterized by rather distinct convex shoulders.

The study plot is located in Central Bohemia in the Czech Republic (N50.457567, E14.508250) (Fig 1a). The research plot is a common agriculture land with no specific nature protection. The sampling was done with agreement of the landlord Mr. Zdeněk Gregor and no plant or animal protected species were involved or endangered. The mean annual precipitation is 588 mm and the mean annual temperature is 8.2°C. The study plot (77 ha) is a part of a larger arable field (176 ha). It is characterized by two perpendicular side valleys (north–south and east–west) connected in the south-west part of the plot. Adjacent slopes reach up to 15°. Large flat upper parts (0–0.5°) occur in the south, north-east and north-west sections of the plot. The mean slope of the plot is 5° (Fig 1b). The plot belongs to a Luvisol region. According to the 1:10 000 soil map from 1960s, Haplic Luvisol dominates the whole plot [42]. The land is in long-term use as arable agriculture land. The large agriculture plot is a result of land consolidation made in the 1950s.

### Terrain sampling

A regular sampling design (30 × 30 m) was used. In total, 111 soil profiles up to a depth of 1 meter were described at each sampling point (Fig 1a). The profile stratigraphy was used for soil classification. Soils with stratigraphy Ap-Bt-C were classified as Luvisols (LU), soil with Ap-C stratigraphy as Regosols (RG) and soils with Ap-Ax-C stratigraphy, where Ap+Ax > 50cm were classified as colluvial soils (CO); for more details see the study of Zádorová et al. [16]. The sampling set was randomly divided to a training (70% points) and validation subset (30% points).

### Digital elevation model

The stereo-photogrammetry based DEM was provided as a 1 × 1 m grid (provider Georeal Ltd.) The DEM was acquired when the field was prepared for seeding (at its minimal roughness). DEM was further smoothed and filtered using a Gaussian filter in SAGA GIS [43]. Six terrain derivatives were used in the analysis. They represent a set of morphometrical attributes that contributed best for soil cover delineation at the study plot in a previous study [16]. Terrain derivatives calculated from the DEM using integrated algorithms implemented in SAGA GIS include: altitude (ALT), slope (SLP), plane curvature (PLANC), topographic position index (TPI), Slope Length and Steepness factor (LS) and convergence index (CONVIN). The original 1 meter grid was resampled to 2, 3, 5, 10, 20 and 30 meters. All the derivatives were calculated for all raster resolutions: 1, 2, 3, 5, 10, 20 and 30 meters (S1 Fig). The terrain derivatives were calculated for the whole arable field and then clipped to the size of the study plot. The range of raster resolution corresponds to the most commonly used raster resolution used for large to medium soil mapping [44].

Produced terrain derivatives were compared using ANOVA, and statistically significant differences between the raster means were assessed by Post hoc LSD test using Statistica 12 [45].

### Spatial prediction models

Machine-learning techniques are widely used in soil prediction mapping [46]. They apply computer-based statistical models, where a fitted model (using training data) is used for prediction purposes on new data [47]. Four models were applied: boosted tree (BT), neural network (NN), random forest (RF) and Classification/Regression Tree (CRT). The delineation of colluvial soils was made for each raster resolution. In total, 28 delineations were calculated. The models were trained using the training subset of sampling points and the models were applied for the whole study plot using the Data miner Recipes in Statistica 12 [45]. The input data were analyzed by “Fast predictor screening” tool [45] to assess the importance of the variables of the predictors and optionally reduce the number of inputs (predictors) and uncover which variables are important for the analysis. The default Statistica software setting was used four chosen models when applied [45].

The models performance was evaluated by widely applied approaches: point validation and visual interpretation of resulting soil maps. Three assessment methods were employed to evaluate the models performance at different raster resolutions: (i) all soil classes (CO, LU, RG) prediction was validated with an independent validation dataset at each resolution, (ii) prediction of only Colluvial soils (CO) was validated with an independent validation dataset at each resolution, (iii) shape and areal extent of the CO soil was examined by visual inspection to assess how well the predicted CO areals are depicted. The validation of the results using the validation dataset was evaluated as overall accuracy.

## Results and Discussion

### Soil survey

**Fig 2:**
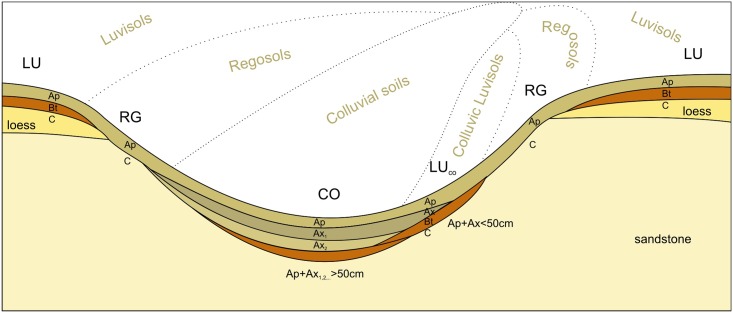
Typical soil toposequence of the study plot. Luvisols (LU) are present on flat or slightly sloping areas (plateau). Regosols (RG) result from accelerated erosion on the steeper convex parts of the landscape. The removed material is transported down the slope and sediments in the base of the side valleys. If the depth of the colluvial material is higher than 50 cm, the soils are classified as Colluvial soils (CO), if less as Colluvic Luvisols (LUco).

According the detailed soil survey, the study plot is formed by Luvisols, Regosols and Colluvial soils. From a total 111 sampling points, 72 were classified as Luvisols, 24 as Regosols and 15 as Colluvial soils. Recent soil development is driven by intensified erosion forming a typical soil mosaic (Fig 2). Luvisols occupy the large gently sloping and flat areas (plateau) with limited erosion and represents a non-degraded soil unit. Regosols are present in the steep convex parts of the plot. Their development occurs by removal of topsoil due to erosion and degradation of the soil profile. Colluvial soils, formed by accumulated humus rich material, with a maximum thickness of 100 cm have developed in the depositional areas of the landscape, represented by two perpendicular side valleys (Fig 1b). In the E-W side valley, the colluvial soil forms a continuous area limited on the sides by back slopes. The N-S side valley is less pronounced with a steeper flow path and the depth of sedimented material forming colluvial soil in north part of the valley, interrupted by a middle part with colluvic Luvisols. The colluvial soils are then found again on the confluence with the E-W side valley. The convex shoulders of the slopes and upper part of back slopes of the side valleys are formed by Regosols. The main gently sopping plateau in the east part of the plot and sloping area in the very south part of the plot are covered by Luvisols.

### Terrain derivatives

**Fig 3:**
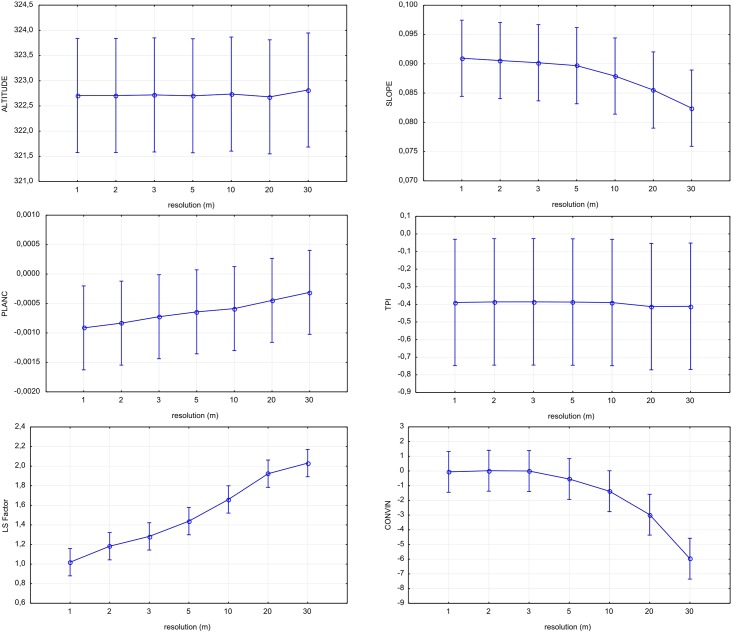
Terrain derivatives means with 95% confidence interval for the different raster resolutions: altitude (ALT), slope (SLP), plane curvature (PLANC), topographic position index (TPI), LS factor (LS) and convergence index (CONVIN).

Rasters of the six terrain derivatives were derived for the given resolutions. Values extracted at sampling points were compared (Table 1). For some properties, clear trends of changing raster means with resolution can be observed (Fig 3). Mean altitude (ALT) does not change with the decreasing raster resolution. It stays constant. This corresponds with other studies, where altitude mean is constant but DEM min/max range decreases with coarser resolution due to the effect of flattening [25]. The mean slope (SLP) was observed to decrease with increasing pixel size, also in concordance with findings of other authors [23]. The LS factor shows a clear trend. With increasing pixel size, mean LS factor increases from 1.02 to 2.03. The trend has a linear shape. The Post hoc LSD test recognises statistically significant differences between some of the resolution (Table 2). Plan curvature has a similar trend as LS factor. The PLANC mean tends from negative values towards 0 with increasing pixel size. The trend has a linear shape, but it is not as steep as for LS factor. The Post hoc LSD test did not show any statistically significant differences between any of the raster resolutions (Table 1). Slope has an opposite trend to LS factor and PLANC; mean slope decreases with increasing pixel size. The trend is weak from 1 to 5 meter resolution and then shows a more significant decrease. Despite a marked trend, the Post hoc LSD test does not prove statistically significant difference between any of the resolution. A mean slope decrease with increasing pixel size was observed by other studies [23] [25] [32] [33] [48] as well. Topographic position index (TPI), as well as ALT, does not change with the decreasing raster resolution and stays constant. Mean convergence index stays constant for 1, 2 and 3 meter raster. CONVIN mean shows values close to zero between 1 and 3 meter resolution and starts to decrease from 5 m raster. The decreasing trend becomes pronounced with the increasing pixel size. The Post hoc LSD test recognises statistically significant differences between some of the resolutions.

**Table 1:** Basic statistic of the terrain derivatives. The table presents mean, median, min., max and standard deviation values of the six terrain derivatives for all resolutions (1, 2, 3, 5, 10, 20 and 30 meters).

Derivatives	Resolution	Mean	Median	Min.	Max.	St.dev.
ALT	1	322,706	322,625	313,960	333,880	5,096
ALT	2	322,708	322,655	313,810	333,890	5,095
ALT	3	322,719	322,645	314,070	333,880	5,092
ALT	5	322,701	322,630	314,070	333,960	5,081
ALT	10	322,736	322,665	313,870	333,830	5,106
ALT	20	322,680	322,445	313,870	333,930	5,031
ALT	30	322,816	322,420	313,620	333,590	5,112
CONVIN	1	-0,059	-0,092	-4,435	10,587	1,623
CONVIN	2	0,018	-0,239	-8,622	30,336	3,908
CONVIN	3	-0,001	-0,259	-12,621	43,191	5,517
CONVIN	5	-0,546	-0,195	-18,303	23,524	4,519
CONVIN	10	-1,374	0,106	-25,923	14,176	5,666
CONVIN	20	-2,974	-2,281	-28,473	17,880	7,269
CONVIN	30	-5,962	-3,000	-40,036	20,336	10,929
LS	1	1,019	0,982	0,023	2,662	0,460
LS	2	1,182	1,120	0,013	2,845	0,512
LS	3	1,283	1,221	0,016	3,083	0,548
LS	5	1,438	1,383	0,015	3,382	0,602
LS	10	1,660	1,643	0,018	3,488	0,687
LS	20	1,923	2,071	0,033	3,319	0,720
LS	30	2,031	2,061	0,167	4,521	0,789
PLANC	1	-0,001	0,000	-0,021	0,009	0,005
PLANC	2	-0,001	0,000	-0,017	0,007	0,004
PLANC	3	-0,001	-0,001	-0,015	0,006	0,004
PLANC	5	-0,001	0,000	-0,010	0,004	0,003
PLANC	10	-0,001	0,000	-0,006	0,004	0,002
PLANC	20	0,000	0,000	-0,004	0,001	0,001
PLANC	30	0,000	0,000	-0,002	0,001	0,001
SLP	1	0,091	0,095	0,009	0,165	0,031
SLP	2	0,091	0,093	0,005	0,165	0,031
SLP	3	0,090	0,093	0,005	0,165	0,031
SLP	5	0,090	0,092	0,004	0,161	0,030
SLP	10	0,088	0,089	0,004	0,159	0,030
SLP	20	0,086	0,089	0,005	0,130	0,027
SLP	30	0,082	0,085	0,016	0,128	0,025
TPI	1	-0,389	0,099	-3,820	1,967	1,591
TPI	2	-0,386	0,160	-3,931	1,983	1,596
TPI	3	-0,386	0,154	-3,776	1,987	1,591
TPI	5	-0,387	0,050	-3,847	1,972	1,593
TPI	10	-0,390	0,042	-3,843	2,040	1,603
TPI	20	-0,413	-0,290	-3,824	1,943	1,563
TPI	30	-0,411	0,149	-4,268	1,983	1,751

**Table 2:** Statistically significant differences between the terrain derivatives means (Post hoc LSD test).

	raster resolution (m)						
	1	2	3	5	10	20	30
ALT	a	a	a	a	a	a	a
SLOPE	a	a	a	a	a	a	a
PLANC	a	a	a	a	a	a	a
TPI	a	a	a	a	a	a	a
LS factor	a	ab	bc	c	d	e	e
CONVIN	a	a	a	a	ab	b	c

### Soil cover delineation

#### Prediction accuracy for whole soil cover

**Fig 4:**
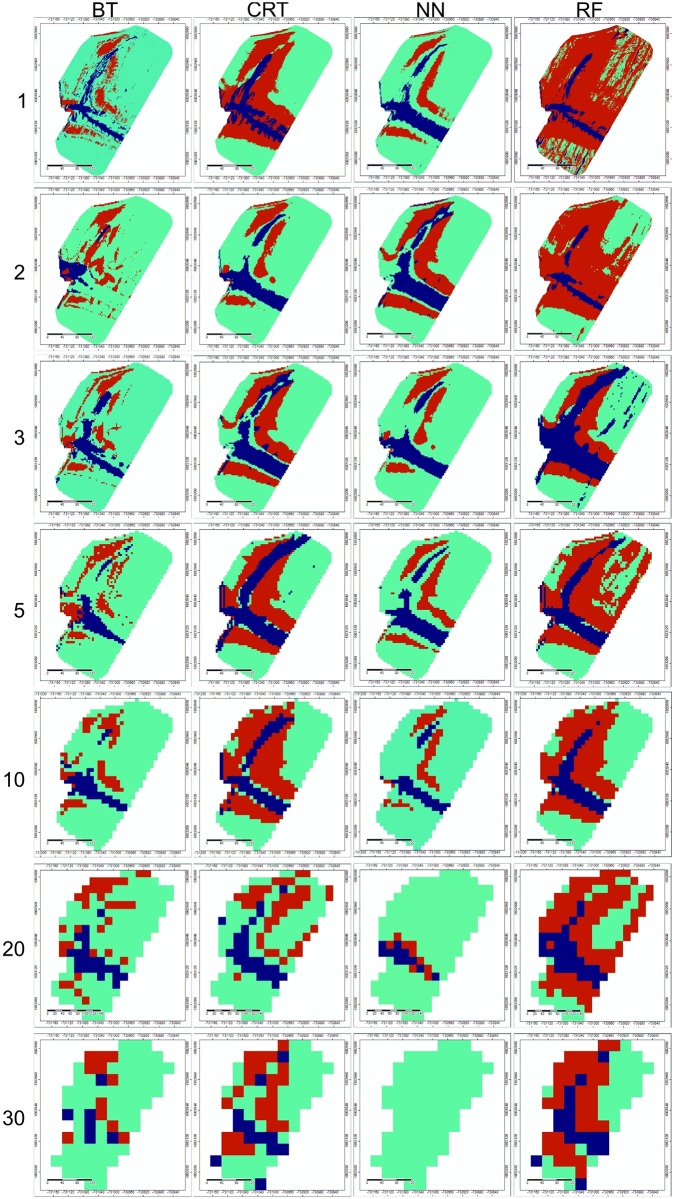
Soil units predicted by the four models at given resolutions 1, 2, 3, 5, 10, 20 and 30 meters. (CO-blue; LU-green; RG-red colour).

**Fig 5:**
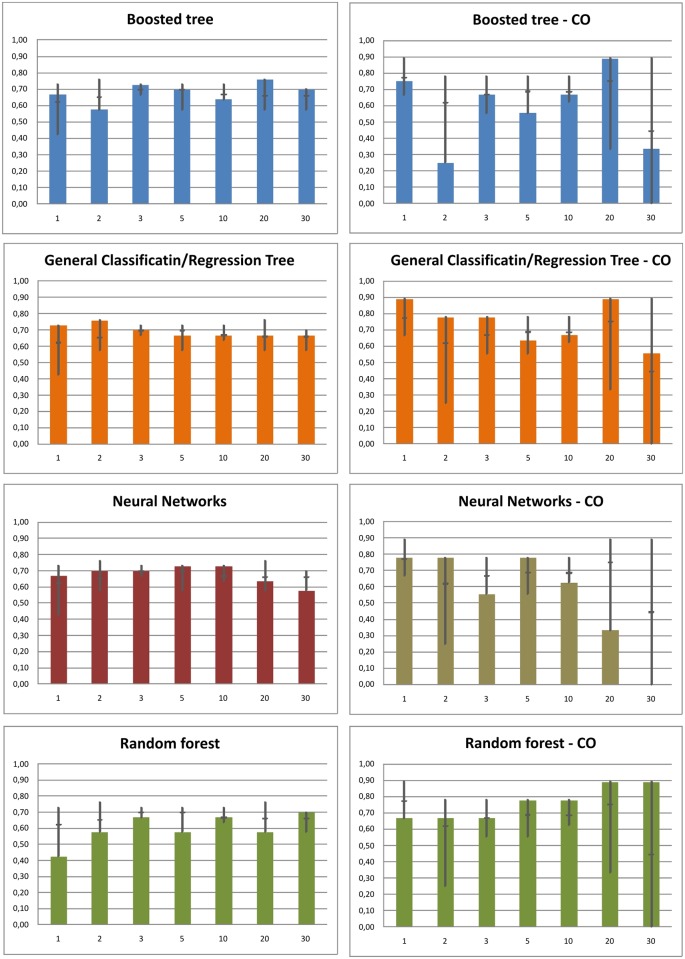
Models accuracy at different raster resolution for all soil classes (left column) and Colluvial soils only (right column). The dark grey vertical line at each column indicates the range (min-max) of all four models at a given resolution. The short horizontal line is the mean value of the four models accuracy.

Soil units were spatially predicted by the four models for all raster resolutions (Fig 4). The performance of the models varies among both models and resolutions (Fig 5). The prediction accuracy ranges between 42% (RF, 1m resolution) and 76% (CRT, 2m resolution). In general, there are only a few trends among the models and resolutions. The BT model does not show any trend in the prediction accuracy among the changing resolution. Best BT model performance was obtained at 20m resolution (73%), when the average accuracy was 67%. CRT model accuracy is highest at 1 and 2m resolution (73% and 76% respectively) and then slightly drops to 67%. NN model reaches best accuracy in the interval between 2 and 10 meters resolution (70–73%). The accuracy clearly decreases to 64 and 58% for 20m and 30m resolution respectively. RF model accuracy is lowest among all the models. Surprisingly, the best accuracy is at 30m (70%) and the worst result was obtained at 1m resolution (42%). Accuracy at other resolutions was less that 70%. When comparing the four models at all resolutions, there is no significant trend in the accuracy of the whole soil cover prediction. At each resolution level, we can find at least one model with the accuracy higher than 70%.

#### Prediction accuracy of Colluvial soils

When we focus on the prediction accuracy of Colluvial soils only, the results differ. In this case, only a match of observed/predicted soil units with colluvial soils is taken into consideration. The models accuracy has a much wider range. The best models accuracy reaches in several cases up to 89%, but the lowest accuracy decreases in one case to 0%. The BT model accuracy is highly unbalanced. The best accuracy was reached at 20m resolution followed by 1m resolution. In contrast, the lowest prediction accuracy was obtained for neighbouring pixel sizes at 2m and 30m respectively. The CRT model provides best prediction accuracy at 1m resolution. With increasing pixel size the accuracy drops to about 55% at 30m resolution. 20m raster represents an exception with prediction accuracy at the same level as in 1m raster. The NN model reaches prediction accuracy maximum at 1, 2 and 5m resolution (78%). Significantly lower accuracy is at 3m resolution (56%). At resolution coarser than 5 meters, a significant trend in accuracy decrease is visible. The prediction accuracy drops to 33% and 0% at 20 and 30m resolution respectively. In contrast to this, RF shows an increasing trend in prediction accuracy with the increasing pixel size. The minimum accuracy is obtained at 1, 2 and 3m resolution (67%) and the maximum at 20 and 30m (78%).

Higher maximal prediction accuracy for Colluvial soil, compared to the prediction accuracy of total soil cover of the study plot, can be explained by choice of terrain derivatives that were in a previous study, evaluated as the best for Colluvial soils differentiation to other soil units [16]. In such a case, the model is not universal, but promotes prediction accuracy for a specific soil unit, in this case the Colluvial soil.

#### Extent and shape of soil units delineation

An important aspect of the model plausibility is the shape and area of the delineated extent of the soil units, especially colluvial soils. Such assessment is of course partly done by the point validation, but visual assessment and interpretation is important as well [34], and it can explain some of the spatial connections of the processes functioning in the landscape. When comparing the results of the modelling with the facts found during the detailed soil survey, we can make several statements.

*Colluvial soil delineation*: In general, the CO soils are well delineated in the more pronounced and wider E-W side valley (Fig 4). There are very few exceptions of this (BT 2, 30m and NN 30m model). The BT model tends to underestimate the area of CO in this part of the study plot when high resolution data is used. The CRT model does not suffer this and keeps the extent of CO soil in the E-W side valley rather constant. NN fails in the CO soils delineation at coarser resolutions (10, 20 and 30m). RF provides a very unbalanced result in this case. It significantly underestimates the CO extent at high resolution (1, 2m), and overestimates the extent at 3m resolution. Surprisingly, lower raster resolutions provide best results. Even at 30 m resolution, where the other methods failed, the CO are still depicted by RF. Partially different model plausibility was assessed in the N-S side valley. In the case of high resolution raster application, the CO are usually well delineated in the N-S side valley. This side valley is shallower and narrower in comparison to the E-W side valley. Therefore, it is more sensitive to changes of the terrain derivatives produced for coarser raster resolutions. This is probably the main reason, why the models at coarser resolution failed to predict the CO in this side valley. Rarely, the CO were overestimated; this happened only in two cases (CRT 5 m and RF 3m).

**Fig 6:**
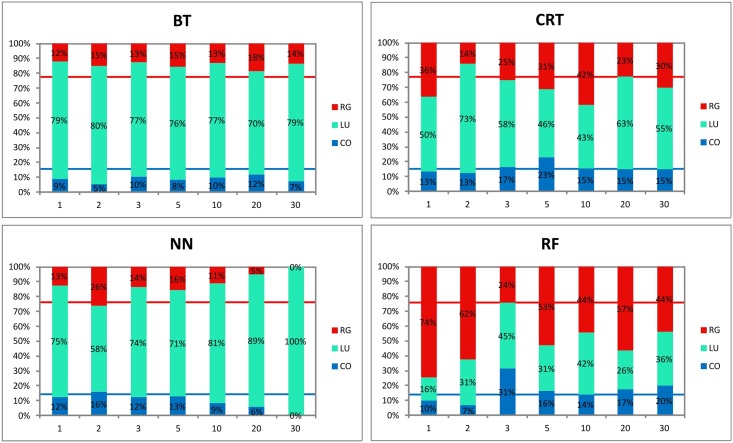
Proportion of soil units on the soil cover of the study plot calculated by the four models at all raster resolutions. The blue and red lines indicate the spatial extent of CO and RG respectively according the soil survey (nr. of observed profiles).

**Fig 7:**
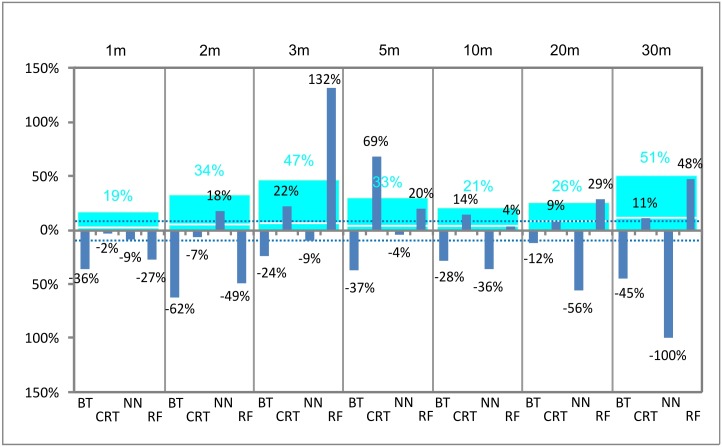
Deviation of CO soils prediction by models from an aerial extent of CO found by the soil survey (relative %). Blue columns show negative and positive relative % deviation. The light blue columns indicate absolute average deviation.

Considering the proportion of CO soils found during the soil survey as a benchmark and comparing it to the proportion of the CO soils calculated by models, several discrepancies can be determined (Fig 6). The CO area represents 14% of the total soil cover according to the soil survey; it reaches lower values in most of the models (17 out of 28 models). The underestimation ranges from 2% to 100% (relative %; Fig 7). The reason of such a discrepancy (as it was already stated above) is, according to visual inspection of predicted maps, mainly the weak CO prediction in the N-S side valley. The lowest average deviation (19%) was determined at 1 m resolution. At all resolutions, we can find at least one model within the accuracy of CO area prediction lower than 10%.

Examining the character of the predicted CO areas in detail, we can observe a significant discontinuity in the CO delineation at 1 meter resolution, forming unrealistic areas of this unit. This is because of an over-detailed resolution where the influence of field roughness is incorporated in the DEM. There are no such features visible at any of the coarser resolutions. Conversely, predicted maps at 20 and 30 m resolution seem to be, from a point of visual interpretation and possible further interpretation of the results, too coarse for practical application in detailed soil mapping. Maps from 2 to 10 m resolution provide reasonable interpretation of the colluvial soil as a part of the cover over the study area.

*Delineation of other soil units*: Delineation of Regosols and Luvisols is even more unbalanced than CO prediction (Fig 6). According to the soil survey, Regosols are present mostly at west and east parts of the N-S side valley, forming a rather narrow strip and occupying about 22% of the study plot. Spatial extent variation is mainly due to the exclusion of Luvisols. In general, the BT model significantly underestimates the RG area on one hand. On the other hand, RG is greatly overestimated by the RF model, which delineates the RG on the majority of the study plot. CRT and NN models provide the most realistic delineation, but none of the models provide consistent results among various resolutions. In the case of BT, CRT and NN models, the predicted RG are logically located at the side valleys, when RF predicts illogically the RG over the plateau where Luvisols are the only unit found by the soil survey.

### General remarks

Our study shows that the pixel/raster resolution ranging from 1 to 30 meters does not significantly influence the model prediction accuracy, contrary to our expectations. When we compare our results with other studies dealing with soil-terrain modelling at similar resolution range, we can find similar results [27] [49]. Some other authors [41] found, that 1 to 20 meter resolution raster can provide very similar results for geomorphological features prediction. There are probably several reasons why the high resolution DEMs with 1 meter or even more detailed resolution do not provide the best results. Excessive accuracy of the high resolution models is stated as one of the reasons [50] [51][52]. The high resolution models often depict instant relief features, such as furrow and other elements, given by cultivation that undesirably influences the general trend of the relief. In our case, we can observe this problem at 1m resolution, when a discontinuous mosaic of Colluvial soils with other units is delineated instead of continuous areas (polygons). The general trend of relief is often considered as a better predictor of long-term soil development. Such results were obtained e.g. for organic layer thickness modelling, when 20 m resolution performed best over a range of 5, 10, 15 and 20 meter raster [53].

On the other hand, too coarse resolution can decrease the modelling accuracy as well. Similarly to our findings, other studies showed that pixel sizes in the range of meters to a few tens of meters (up to 20–25 m) provide satisfactory results, while the prediction accuracy decreases at coarser resolution. Several studies [31] [32] [33] [54] give 30(50)– 100 m resolution as an upper limit of pixel size in the soil-terrain modelling. Beyond these limits, the relief details depiction is insufficient to properly represent soil properties and processes development and distribution, resulting in the disappearance of fine-scale features [33]. The influence is more significant in areas with more dissected relief [34].

Therefore, a proper raster resolution should be selected according to the aim of the modelling and character of the study area, because the influence of raster resolution on modelling accuracy differs, as well locally according to local settings [51].

In our study, the use of machine-learning algorithms resulted in very different outputs. Such a result is common in many other studies. There are recommendations from authors [47] who tested a larger set of machine learning models to not restrict to a single method or a small selection of them, but to apply a wider range of models.

## Conclusions

- The purpose of our study was to test the influence of a raster resolution on colluvial soils delineation by predictive mapping using soil terrain modelling. Colluvial soils are an important part of many of the agriculturally used regions. They are a result of sedimentation of eroded material in concave parts of the landscape. We tested the interaction between the pixel resolution (1, 2, 3, 5, 10, 20 and 30m) and the prediction accuracy of four models based on machine learning techniques. We evaluated the plausibility of applied models by both independent validation point dataset and visual evaluation of predicted delineations of colluvial soils.
- Six terrain derivatives—altitude (ALT), slope (SLP), plane curvature (PLANC), topographic position index (TPI), LS factor (LS) and convergence index (CONVIN)- were calculated from the 1 m DEM at resolution 1, 2, 3, 5, 10, 20 and 30 meters. Some of the derivatives showed a trend of changing raster means with changing resolution. However, no statistically significant difference between the raster resolutions was found in most cases.
- The study shows, that the pixel/raster resolution ranging from 1 to 30 meters does not significantly influence the model prediction accuracy, contrary to our expectations. The models accuracy shows high variability. No significant general trend in the whole soil cover prediction accuracy was determined when comparing all four models at all resolutions. At each resolution level, we can find at least one model of which accuracy exceeds 70%.
- Higher maximal prediction accuracy for Colluvial soil, compared to the prediction accuracy of total soil cover of the study plot, can be explained by the choice of terrain derivatives that were evaluated as the best for Colluvial soils differentiation to other soil units. The resulting improved CO prediction accuracy weakens the versatility of the model.
- The shape of the colluvial soil area was visually evaluated. The most reasonable interpretation was provided by intermediate resolutions (from 2 to 10 meters). Finer resolution produced discontinuous areas, reflecting the details of instant relief features, while the coarser resolution ignored important details reflecting the soil cover development.
- Similarly to our study, many other studies showed that pixel sizes in the range of meters to a few tens of meters (up to 20-25m) provide satisfactory results, and that the prediction accuracy decreases with coarser resolution.

## Supporting Information

S1 Fig Calculated terrain derivatives for the study plot. The six terrain derivatives (altitude (ALT), slope (SLP), plane curvature (PLANC), LS factor (LS), convergence index (CONVIN) and topographic position index (TPI)) were calculated for 1, 2, 3, 5, 10, 20 and 30 DEM resolution in SAGA GIS.
(TIF)
