# Supplementary figures and images for: Influence of Elevation Data Resolution on Spatial Prediction of Colluvial Soils in a Luvisol Region
Source: PLoS One. 2016 Nov 15;11(11):e0165699. doi: 10.1371/journal.pone.0165699 (PMC5112918; doi:10.1371/journal.pone.0165699)

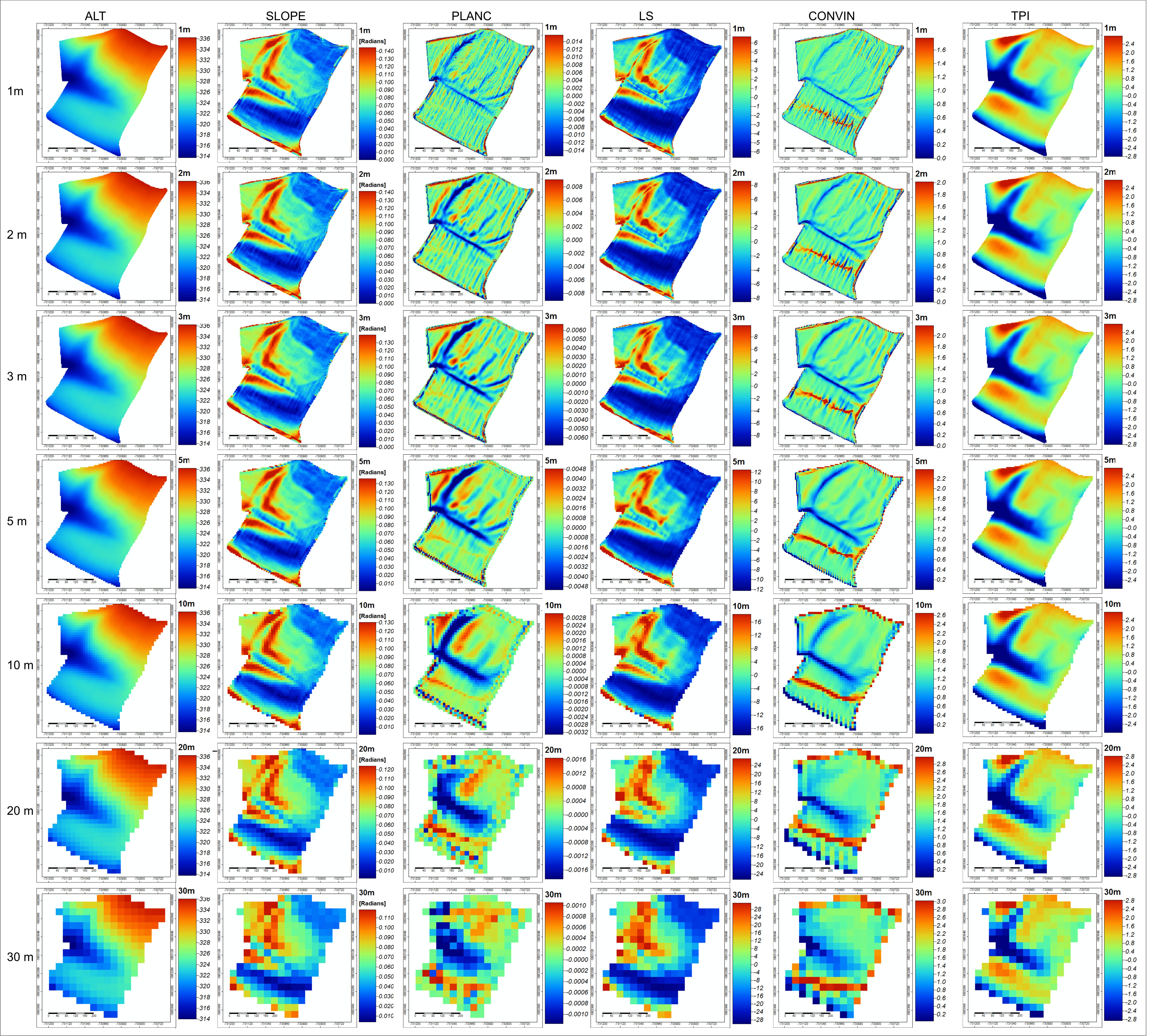

Supplement: S1 Fig — The six terrain derivatives (altitude (ALT), slope (SLP), plane curvature (PLANC), LS factor (LS), convergence index (CONVIN) and topographic position index (TPI)) were calculated for 1, 2, 3, 5, 10, 20 and 30 DEM resolution in SAGA GIS. (TIF) [file pone.0165699.s001.tif]
